# Are independent mobility and territorial range associated with park visitation among youth?

**DOI:** 10.1186/1479-5868-11-73

**Published:** 2014-06-09

**Authors:** Jenny Veitch, Alison Carver, Clare Hume, David Crawford, Anna Timperio, Kylie Ball, Jo Salmon

**Affiliations:** 1 Centre for Physical Activity and Nutrition Research, Deakin University, 221 Burwood Highway, Burwood, VIC 3125, Australia

**Keywords:** Park visitation, Adolescents, Youth, Active transport, Independent mobility, Territorial range

## Abstract

**Background:**

Parks are important for providing opportunities for physical activity among youth. Apart from engaging in physical activity whilst visiting a park, active transportation (e.g. walking or cycling) to parks is potentially an additional source of physical activity. Previous research has shown that a major barrier to young people visiting parks is their inability to visit parks unaccompanied by an adult. It is not known; however, whether young people who have greater independent mobility and territorial range (ability to move around their neighbourhood alone or with friends, unaccompanied by an adult) are more likely to visit parks. This study examined park visitation and travel mode to parks and whether independent mobility and territorial range were associated with park visitation among youth living in disadvantaged areas of Victoria, Australia.

**Methods:**

In 2010–11, 311 youth aged 8–16 years self-reported their park use, active transport, independent mobility to parks, and territorial range. Logistic regression models determined the odds of park visitation (once per week or more) according to independent mobility and territorial range, adjusting for key covariates.

**Results:**

Overall, 75% of participants reported visiting parks, and 37% visited their ‘usual’ park at least once per week. Of those who reported visiting parks, 87% travelled to the park they usually visited using active transport: 57% walked, 22% cycled, and 8% used a scooter/skateboard. Just 15% and 13% of youth regularly walked or cycled alone to parks/playgrounds respectively, and 25% and 19% regularly walked or cycled with friends or siblings (no adults) respectively. For the 84% who reported having parks/playgrounds within walking distance from home, those who regularly walked alone to parks (OR 3.61; CI=1.67, 7.80), and regularly walked (OR 2.27; CI=1.14, 4.55) or cycled (OR 3.38; CI=1.73, 6.62) with friends to parks, were significantly more likely to visit a park at least once per week, compared to others.

**Conclusions:**

This study showed that active transport is frequently used by this sample of young people to travel to parks. Findings also highlight the potential importance of providing opportunities for youth aged 8–16 years to visit local parks independent of an adult.

## Background

Public parks are important for providing opportunities for physical activity among youth [1] and for supporting initiatives to reduce the prevalence of obesity in young people [2-4]. Studies have shown positive associations between objectively measured availability of local parks and playgrounds and young people’s physical activity [5,6]. Parent proxy-report and youth self-report perceptions of living within close proximity to parks have also been associated with increased likelihood of being active within a park among children and adolescents [7,8] and with engaging in more moderate- to vigorous-intensity physical activity among adolescents [8]. Enhancing opportunities for youth to visit parks is a promising strategy for promoting physical activity. Park use may be particularly advantageous for increasing physical activity levels in low socioeconomic status (SES) neighbourhoods where residents are at an increased risk of inactivity and associated poor health [9].

Independent mobility refers to young people’s freedom to move around their neighbourhood without adult accompaniment [10]. Youth with greater independent mobility tend to spend more time walking or cycling around their neighbourhood to reach places and generally also have greater ‘territorial range’, meaning that they are permitted to roam further from home and visit a broader range of destinations without adult accompaniment [11]. Previous research has shown that a major barrier to young people visiting parks is their inability to visit parks unaccompanied by an adult [12,13]. We are unaware, however, of any research that has examined whether young people who have greater independent mobility and territorial range are more likely to visit parks.

Apart from engaging in physical activity whilst visiting a park, active transportation (e.g. walking or cycling) to parks is potentially an additional source of physical activity among youth. Few studies have examined the mode of transport young people use to travel to the parks they usually visit. A study [7] in the USA found that walking and cycling to recreational sites including small and large parks was associated with frequent active use of these sites by young people [7]. However, that study did not examine whether travel to the parks was performed independent of an adult.

While access to parks from home is important for active transport to be a viable option, in low socioeconomic status (SES) neighbourhoods, youth often travel more than twice the distance of youth in higher SES areas to reach the park they usually visit [14]. This may make it more difficult for youth living in low SES neighbourhoods to use active transport to access the parks they visit. Few studies have examined park visitation or use of active transport (particularly independent of an adult) to reach the park among youth living in low SES neighbourhoods. The aims of this study were therefore to examine the frequency of park visitation and travel mode to parks among a sample of youth living in low SES areas, and to investigate whether independent mobility and territorial range on their own or with friends/siblings (unaccompanied by an adult) were associated with park visitation.

## Methods

Participants for this study were recruited from the Resilience for Eating and Physical Activity Despite Inequality (READI) study, a longitudinal cohort study examining resilience to obesity among socio-economically disadvantaged women and children. The methods have been described in more detail elsewhere [15]. Data collection was conducted in October 2010 - June 2011. Ethics approval was granted by the Deakin University Human Ethics Advisory Group, the Catholic Education Office and the Department of Education and Early Childhood Development.

### Procedure and participants

Briefly, women aged 18-45 years residing in 40 urban and 40 rural low SES areas of Victoria, Australia were invited to complete a postal survey regarding their dietary and physical activity behaviours. Disadvantaged areas were randomly selected from suburbs in the bottom tertile of the Victorian Socio-Economic Index for Areas (SEIFA) distribution [16] within urban and rural strata. Of these women (n = 11,940) who were randomly sampled from the electoral roll, 4934 consented to participate (41% response rate). Women (n = 1457) with a child aged between 5–12 years were invited to complete a further questionnaire on their child’s health behaviours. In total, data were collected for 636 children (44% of eligible children). Baseline data collection was conducted between August 2007 and July 2008. In August 2010, women and children were invited to participate in this three-year follow-up study nested within the READI study, to examine adolescents’ active transport and independent mobility. Parental consent to participate in the current study was obtained for 311 children/adolescents (henceforth called adolescents), who comprised 49% of the original sample. Participants aged 8–16 years completed a survey at school (88%) or home (12%) about their park use, active transport and independent mobility to parks in their local neighbourhood, and their territorial range from home. During survey completion a research assistant was available to assist participants understand and respond to any questions where required. Participants were provided with a small ball as compensation for their time.

### Measures

#### *Park usage*

Adolescents were asked whether or not they visited parks and if so, which park they usually visited and how often they usually visited this park. Response options included: ‘most days’; ‘once per week’; ‘several times per month’; ‘once per month’; ‘less than once per month’; and ‘have not visited in past 6 months’. Responses were dichotomised as ‘visit at least once per week’ or ‘not’.

#### *Travel mode to parks/playground*

Adolescents were asked to report by ticking the most appropriate response, how they typically travelled to the park that they usually visited. Response options included: ‘walk’; ‘ride a bike’; ‘skateboard/scooter/rollerblades’; and ‘car’.

#### *Independent mobility to parks*

Participants were asked if any parks/playgrounds were located within walking and/or cycling distance from their home. Those who reported living within walking/cycling distance of a park were then asked how often they usually walked to nearby parks: (a) by themselves; (b) with adult accompaniment; and (c) with friends/siblings (no adults). Response options included: ‘never’; ‘rarely’; ‘sometimes’; ‘often’; and ‘very often’. Two dichotomous variables were derived from these responses to indicate (1) whether participants regularly (often or very often) walked alone to parks (or not) and (2) whether they regularly (often or very often) walked with friends/siblings (no adults) to parks (or not). Adolescents were asked the same questions in relation to frequency of cycling to nearby parks/playgrounds. One week test-retest reliability for these variables was established in a separate study of 48 children aged 8–9 years in 2010 and ranged from moderate (κ = 0.40) to perfect (κ = 1.0) in the case of cycling without adult accompaniment.

#### *Territorial range*

Adolescents reported how far from home they were allowed to roam on their own. Response options included: ‘I am not allowed out alone’; ‘within my street’; ‘within 2–3 streets away from home’; ‘within 15 minutes’ walk from home’; and ‘more than 15 minutes’ walk from home’. This question was repeated in relation to how far from home they were allowed to roam with friends (unaccompanied by an adult). For each of these variables, responses were dichotomised to indicate whether or not they were allowed to roam more than 15 minutes from home. Test-retest reliability for these variables was moderate (κ = 0.59; 0.52 respectively).

#### *Demographics*

Mothers reported their highest level of education which was collapsed into three categories: low = <12 years of schooling (no formal education or year 10/equivalent); medium = 12 years of schooling (having completed year 12 or equivalent, a trade/apprenticeship, or certificate/diploma); and high = >12 years of schooling (having a university or higher university degree). Mothers also reported their car ownership, and their child’s age and sex.

## Data analyses

Analyses were conducted using SPSS v21 and Stata SE v12. Descriptive analyses on the dependent and independent variables were performed. Among those living within walking/cycling distance to a park, crude logistic regression models adjusting for clustering by suburb were conducted to examine the odds of visiting their usual park at least once per week according to independent mobility to the park and territorial range. Adjusted logistic regression models controlling for sex, age, urban/rural location and clustering by suburb were also performed. Potential interactions of explanatory variables with sex and age were examined.

## Results

Three-hundred and eleven adolescents completed the survey. The mean age was 12.2 years (range 8–16 years), 55% were attending primary school (first seven years of schooling in Australia), 45% were boys and 70% resided in rural areas. Almost one-third of mothers had completed more than 12 years of education, and 99% of all households had access to a car (Table 1).

**Table 1:** Characteristics of study sample (2010–11 Victoria, Australia)

	
Characteristics of the child	
Sex (% boys)	44.7
Age (years; mean (SD))	12.2 (2.19)
Attending primary school (%)	55.0
Resides in rural area (%)	69.5
Maternal education (%)	
<12 years	20.2
12 years	46.4
>12 years	33.4
Frequency of visitation to usual park visited^a^ (%)	
Most days	15.5
Once per week	21.4
Several times per month	32.2
Once per month	10.3
Less than once per month	14.2
Have not visited in past 6 months	6.44
Usual mode of transport used when visiting park^a^ (%)	
Car	13.0
Walking	57.1
Cycling	21.6
Scooter/skateboard	8.2
Independent mobility to parks (%)	
Regularly walks^b^ alone to parks/playgrounds	15.3
Regularly walks^b^ with friends or siblings (no adults) to parks/playgrounds	24.8
Regularly cycles^c^ alone to parks/playgrounds,	13.2
Regularly cycles^c^ with friends or siblings (no adults) to parks/playgrounds	18.8
Territorial range (%)	
Allowed to roam >15 minutes from home alone	37.0
Allowed to roam >15 minutes from home with friends	49.5

^a^Among those who visit park(s) (n = 233).

^b^Among those who reported that a park/playground was within walking distance from home (n = 262).

^c^Among those who reported that a park/playground was within cycling distance from home (n = 272).

### Park visitation

A total of 233 adolescents (75%) reported visiting parks. Among these, 37% reported visiting their usual park at least once per week and 69% visited at least several times per month. Of those who reported visiting parks, 87% travelled to the park they usually visited using active transport: 57% walked, 22% cycled, and 8% used a scooter/skateboard (Table 1).

Of the 311 adolescents, 262 (84%) reported that parks/playgrounds were within walking distance from home. Of these, less than one in four regularly walked or cycled independently to parks/playgrounds. Just over one-third of all participants reported that they could roam more than 15 minutes from home alone and 50% reported that they could roam more than 15 minutes from home with friends (Table 1).

With respect to independent mobility, Table 2 shows that after adjusting for age, sex, urban/rural location and clustering by suburb, youth who reported regularly walking or cycling to parks without adult accompaniment were more likely to visit parks at least once per week. For example, the odds of visiting parks at least once per week were more than three-and-a-half times as high among participants who regularly walked alone to parks, compared with those who walked alone to parks less regularly. Furthermore, the odds of visiting parks at least once per week was more than two times higher among youth who regularly walked with friends (no adults) to parks and more than three times higher among those who regularly cycled with friends (no adults) to parks, when compared with adolescents who did not do this regularly. Territorial range was not significantly associated with frequency of park visitation. No significant interactions with age and sex were identified (data not shown).

**Table 2:** Associations between independent mobility to parks, territorial range and visiting a park at least once per week* (2010–11 Victoria, Australia)

	**Visiting a park at least once per week**^**a**^			
	**Unadjusted models**^**b**^		**Adjusted models**^**c**^	
	**OR (95% CI)**	**p**	**OR (95% CI)**	**p**
Independent mobility				
Regularly walks alone to parks	3.31 (1.61, 6.81)	**0.001**	3.61 (1.67, 7.80)	**0.001**
Regularly walks with friends (no adults) to park	1.72 (0.88, 3.36)	0.110	2.27 (1.14, 4.55)	**0.020**
Regularly cycles alone to parks	2.35 (1.05, 5.26)	**0.038**	2.10 (0.85, 5.21)	0.110
Regularly cycles with friends (no adults) to park	2.96 (1.54, 5.69)	**0.001**	3.38 (1.73, 6.62)	**<0.001**
Territorial range				
Allowed to roam >15 mins from home alone	1.32 (0.76, 2.30)	0.319	1.47 (0.80, 2.71)	0.217
Allowed to roam >15 mins away from home with friends	1.20 (0.71, 2.04)	0.491	1.50 (0.79, 2.82)	0.213

^a^Odds are for visiting a park at least once per week compared to visiting a park less than once per week.

^b^Logistic regressions without adjustment for covariates, controlling for clustering by suburb.

^c^Logistic regression controlling for sex and age, urban/rural location and clustering by suburb.

*Includes only participants who reported that parks/playgrounds were within walking (n = 262) or cycling (n = 272) distance from home.

Significant associations are shown **in bold**.

OR = Odds Ratio; 95% CI = 95% Confidence Interval.

## Discussion

This study is novel as it is one of the first to examine how independent mobility and territorial range were associated with frequency of park visitation among a sample of adolescents living in low SES urban and rural areas. We found that the majority of our sample visited parks, with more than one-third visiting at least once per week. Active transport was the most popular mode of transport used to access the park. These findings are consistent with previous research which found that among a sample of 1859 British children (mean age 10 years) 82% usually travelled by walking or cycling when visiting the park [17]. Few other studies have examined the mode of transport young people use to visit parks; however, a recent systematic review of studies involving young people aged 3–18 years showed that active transport to destinations other than school was positively associated with physical activity [18]. Travelling to parks using active modes is likely to be an important source of physical activity among young people, particularly among youth living in low SES areas and is an important next step to examine in future research.

Territorial range was not significantly associated with likelihood of visiting a park once or more per week. This was an interesting finding as, to date, most research on territorial range has focused on sex differences and on age-related increases [19,20] and has not examined children’s territorial range in relation to park visitation (e.g. whether children who are allowed to roam further from home without adult accompaniment visit parks that are within walking distance of home more frequently).

The current study found that youth who reported regularly walking or cycling to parks without adult accompaniment were more likely to visit parks at least once per week. To our knowledge, no previous studies have specifically examined associations between independent mobility and park visitation; however, previous research has shown that children with greater independent mobility are more active [18] and spend more time playing outdoors [21,22]. For example, in an Australian study, children aged 10–12 years who reported that they were allowed to walk on their own near home were more than two and a half times as likely to spend time playing outdoors after school compared with children who were never allowed to walk on their own [21]. In a UK study among 1307, 10–11 year old children, greater independent mobility was associated with an increased likelihood of boys playing outdoors every day [22]. In these two previous studies, activity and independent mobility were self-reported and the location of the outdoor play was not specified and may have included various locations such as the yard at home, the street and/or the local park.

Although not examined in the current study, previous research has reported that road safety and ‘stranger danger’ appear to be major causes of parental anxiety in relation to their child’s safety in the neighbourhood [23]. These concerns may cause parents to restrict their children’s outdoor play, active transport and independent mobility. A recent study among adults in the US found that participants were more likely to visit local parks if they did not have to cross or travel on a high traffic speed road between home and their closest park [24]. Although this study was among adults, it would be reasonable to argue that the effects of traffic concerns may be even more detrimental to independent mobility and park visitation among youth. Conversely, features within the neighbourhood built environment, such as the presence of green spaces [25], outdoor play spaces [26] and housing density [27] may promote children’s independent mobility. In order to increase opportunities for youth to access neighbourhood destinations such as parks independently of an adult, it is important to further investigate strategies to address these barriers/facilitators to increase opportunities for independent mobility.

The findings from this study are limited by the cross-sectional study design which precludes causal inference, and generalisability may be limited to those living in disadvantaged areas. Public transport was not included as an option for means of transport to visit parks and this is a limitation as public transport may involve active transport (i.e. walking to a bus stop) and may also affect independent mobility. The relatively small sample size also restricted our ability to stratify by age, sex or urban/rural residence. Participants ranged in age from 8–16 years and it is possible that park use and independent mobility varies across these ages. Further, data collection was carried out over a nine month period between October 2010 and June 2011 and although participants were asked to report their usual participation, some of the respondents’ answers may have been influenced by the weather conditions at the time they completed the survey. In addition, the response categories for the item on park visitation did depend on participants’ interpretation of ‘several times’ but this is not atypical and it is not possible to determine how this may have affected the results. Finally, when examining independent mobility to parks, only including those who reported having a park/playground within walking distance from home may have reduced heterogeneity in the distance travelled to visit the park. It is also important to acknowledge the use of self-report, although guided interviews strengthened this methodology. Additional strengths included the recruitment of youth in rural as well as urban areas and the focus on low SES neighbourhoods since this population group has been under-researched.

## Conclusions

This study showed that active transport was frequently used by the young people in this sample to travel to parks and highlights the potentially important role of independent mobility for increasing park visitation among youth aged 8–16 years. In order to promote active transport and independent mobility to parks it is important for planners to site local parks within walking/cycling distance of the majority of homes or within residential areas. Further research is also required to better understand the factors that may restrict young people’s independent mobility and how these concerns can be minimised to enhance opportunities for young people to visit local parks independently of an adult.

## Competing interest

The authors declare that they have no competing interests.

## Authors’ contributions

JV conducted the data analyses and drafted the manuscript. AC assisted with the data analyses and manuscript writing. CH, DC, AT, KB, and JS provided critical comment on the analyses and the manuscript. All authors contributed to the drafting of the manuscript and read and approved the final manuscript.
